# Association of Serum ELMO-3 Levels with Metastatic Status and Survival Outcomes in Non-Small Cell Lung Cancer

**DOI:** 10.3390/cimb48040427

**Published:** 2026-04-21

**Authors:** Hilal Oğuz Soydinç, Murat Serilmez, Ceren Tilgen Yasasever, Elif Bilgin Doğru, Uğur Gezer, Şule Karaman, Nergiz Dağoğlu Sakin, Derya Duranyıldız

**Affiliations:** 1 Department of Basic Oncology, Oncology Institute, Istanbul University, 34093 Istanbul, Türkiye; murat.serilmez@istanbul.edu.tr (M.S.); ceren.yasasever@istanbul.edu.tr (C.T.Y.); elif.bilgin@istanbul.edu.tr (E.B.D.); ugurd@istanbul.edu.tr (U.G.); deryady@istanbul.edu.tr (D.D.); 2 Department of Clinic Oncology, Oncology Institute, Istanbul University, 34093 Istanbul, Türkiye; sule.karaman@istanbul.edu.tr (Ş.K.); ndagoglu@istanbul.edu.tr (N.D.S.)

**Keywords:** cell motility, ELMO-3, metastasis, non-small cell lung cancer, prognostic factors, serum biomarker

## Abstract

Non-small cell lung cancer remains one of the leading causes of cancer-related mortality worldwide, and identifying molecular markers associated with tumor progression and metastasis is important for improving patient management. This study investigated serum ELMO-3 levels in patients with NSCLC and evaluated their relationship with clinicopathological characteristics. Serum samples from 50 NSCLC patients and 20 healthy controls were analyzed. ELMO-3 concentrations were measured using an enzyme-linked immunosorbent assay. Statistical analyses included non-parametric group comparisons, receiver operating characteristic curve analysis, Kaplan–Meier survival analysis, and multivariate Cox proportional hazards regression. The mean ELMO-3 level was 0.409 ± 0.543, which was used as the cutoff value to categorize patients into low- and high-ELMO-3 groups; 76% of patients were classified as low-ELMO-3 and 24% as high-ELMO-3. The results showed that serum ELMO-3 levels did not differ significantly between NSCLC patients and healthy controls and were not associated with metastatic status. However, a significant association was observed between ELMO-3 expression status and tumor histopathology. Survival analysis demonstrated that distant metastasis and radiotherapy were significantly associated with overall survival. In multivariate analysis, age, operability, distant metastasis, and serum ELMO-3 levels were identified as independent factors associated with survival. These findings suggest that circulating ELMO-3 may have potential prognostic relevance; however, the results should be interpreted with caution and require validation in larger, independent cohorts.

## 1. Introduction

Lung cancer continues to represent the leading cause of cancer-related mortality worldwide and remains a major global health problem [1]. Lung cancer is generally classified into three main subtypes, of which non-small cell lung cancer (NSCLC) accounts for approximately 85% of all cases [2]. Despite considerable advances in diagnostic and therapeutic approaches, the prognosis of patients with NSCLC has remained poor. This has largely been attributed to the fact that most patients are diagnosed at advanced stages, frequently after the development of metastatic disease, together with the limited availability of effective therapeutic options [3]. Metastasis has therefore been recognized as the primary cause of mortality among patients with NSCLC. Consequently, the identification of molecular factors associated with metastatic progression has been considered crucial for improving patient monitoring and risk stratification [4]. Metastatic progression refers to the multistep biological process by which tumor cells disseminate from the primary tumor, invade surrounding tissues, enter the circulation, and establish secondary tumors at distant organs [5].

Molecules involved in cell migration and cytoskeletal remodeling have been reported to play critical roles in tumor invasion and metastatic dissemination [6]. Members of the Engulfment and Cell Motility (ELMO) protein family have been identified as key regulators of cellular motility [7]. ELMO3 is a protein that regulates phagocytosis of apoptotic cells and cell migration by interacting with DOCK1, thereby promoting engulfment processes and mediating cell shape changes [8]. Tumor invasion and metastasis have been shown to be strongly influenced by molecular pathways regulating cytoskeletal reorganization, cellular motility, and cell–matrix interactions. ELMO proteins have been described as important mediators of cytoskeletal dynamics and cell migration through their interaction with signaling molecules involved in actin cytoskeleton remodeling. Among this protein family, ELMO-3 has been reported to be associated with tumor cell motility, invasion, and metastatic potential in several malignancies. In the literature, ELMO-3 expression has been investigated in various cancer types, including salivary gland [9], laryngeal [10], gastric [11], head and neck squamous cell [12], colorectal cancers [13,14], and glioblastoma [15]. Previous studies have indicated that alterations in ELMO-3 expression may enhance cellular migration and invasion, thereby contributing to tumor progression and metastatic spread. However, only a limited number of studies have evaluated the role of ELMO-3 in non-small cell lung cancer [16,17,18]. Moreover, the clinical significance of circulating ELMO-3 levels in patients with NSCLC has remained largely unclear.

Circulating biomarkers have increasingly been recognized as valuable tools for the minimal non-invasive diagnosis, monitoring, and prognostic assessment of cancer [19]. The evaluation of serum levels of molecules involved in tumor invasion may provide important insights into metastatic potential and disease progression [20]. In this context, the investigation of serum ELMO-3 levels may contribute to clarifying its potential role as a biomarker associated with metastatic status and clinical outcomes in NSCLC. Therefore, the present study was designed to investigate serum ELMO-3 levels in patients with NSCLC and to evaluate their association with metastatic status, clinicopathological characteristics, and survival outcomes. It was hypothesized that serum ELMO-3 levels might be associated with metastatic progression and could potentially serve as a non-invasive biomarker for predicting disease behavior in patients with NSCLC.

## 2. Materials and Methods

### 2.1. Patients and Sample Collection

This study was carried out using archived serum samples obtained from 50 patients diagnosed with NSCLC. In addition, a control group consisting of 20 healthy volunteers with comparable age and gender distribution and no history of malignancy was included. The study protocol involving human-derived materials was approved by the institution’s ethics committee. All procedures were conducted in accordance with the principles of the Declaration of Helsinki, and written informed consent was obtained from all participants after they had been informed about the purpose and scope of the study.

Tumor staging was determined according to the guidelines of the American Joint Committee on Cancer (AJCC) and the Union for International Cancer Control (UICC). Operability was defined as the surgical resectability of the tumor, determined based on tumor stage, patient performance status, and multidisciplinary clinical evaluation according to established oncological guidelines. Approximately 5 mL of peripheral venous blood was collected from each participant into biochemical collection tubes containing a gel clot activator (BD Vacutanier SST II Advance, Becton, Dickinson and Company, Franklin Lakes, NJ, USA). After collection, serum samples were centrifuged (Hettich Universal 32, Kirchlengern, North Rhine-Westphalia, Germany) at 3000× *g* for 10 min to allow proper separation of the serum fraction. The obtained serum was then carefully transferred into sterile microcentrifuge tubes, aliquoted to avoid repeated freeze–thaw cycles, and stored at −80 °C until further biochemical analyses were performed.

### 2.2. Determination of ELMO-3 by Enzyme-Linked Immunosorbent Assay (ELISA)

ELMO-3 concentrations were measured using a commercially available photometric assay kit (Yehua Biological Technology Co., Ltd., Shanghai, China) according to the manufacturer’s protocol. Optical density (OD) values from the samples were interpreted using the standard curve. The curve was constructed by plotting the mean OD values of each standard concentration and fitting them with the most appropriate regression model. Sample concentrations were determined by interpolating their OD values from the standard curve. Assay precision was evaluated by calculating intra-assay and inter-assay coefficients of variation (CV%), which were below 8% and 10%, respectively.

### 2.3. Statistical Analysis

All statistical analyses were performed using IBM SPSS Statistics software (version 31; IBM Corp., Armonk, NY, USA). Descriptive statistics were presented as mean ± standard deviation for continuous variables and as frequencies and percentages for categorical variables. Median, minimum, and maximum values were also reported where appropriate. The normality of continuous variables was evaluated using the Kolmogorov–Smirnov test. Since ELMO-3 levels were not normally distributed (*p* < 0.05), non-parametric tests (Mann–Whitney U and Kruskal–Wallis tests) were used for comparisons between groups. For categorical variables, associations between clinicopathological parameters and ELMO-3 expression status (low vs. high) were assessed using the chi-square test. When expected cell counts were small or when variables contained more than two subgroups, Monte Carlo simulation was applied to obtain more reliable *p* values. Receiver operating characteristic (ROC) curve analysis was performed to evaluate the diagnostic performance of serum ELMO-3 levels in distinguishing between patient and control groups, as well as metastatic and non-metastatic patients. The diagnostic accuracy was assessed by calculating the area under the curve (AUC) with corresponding 95% confidence intervals (CI). Survival analyses were conducted using the Kaplan–Meier method, and differences between survival curves were evaluated using the log-rank test. Overall survival (OS) and progression-free survival (PFS) were analyzed according to clinicopathological and treatment-related variables. To identify independent prognostic factors associated with survival outcomes, multivariate analysis was performed using the Cox proportional hazards regression model. Variables included in the multivariate model comprised age, gender, smoking status, COPD, ECOG performance status, histopathology, tumor size, operability, radiotherapy, chemotherapy, recurrence, distant metastasis, and serum ELMO-3 levels. Hazard ratios (HR) and 95% confidence intervals (CI) were calculated for each variable. A *p* value < 0.05 was considered statistically significant.

## 3. Results

The clinical and demographic (gender, age, and smoking) characteristics of 50 patients with NSCLC and 20 healthy individuals were evaluated. Of the patients, 56% (28/50) were older than 60 years, and 44% (22/50) were younger than 60 years. Most cases were male (84%, 42/50), while females accounted for 16% (8/50). A history of smoking was present in 86% of patients (43/50), while 14% (7/50) had no smoking history. Healthy groups were age- and gender-matched with NSCLC patients. Smoking was reported in 50.4% of healthy group and none had upper respiratory tract infections. Chronic obstructive pulmonary disease (COPD) was detected in 28% (14/50), and 72% (36/50) had no COPD. Regarding performance status, 90% (45/50) were classified as ECOG I–II, and 10% (5/50) as ECOG III–IV. According to TNM staging, 38% (19/50) were in the early stage, and 62% (31/50) were in the advanced stage. Histopathological evaluation showed that adenocarcinoma was the most common tumor type (80%, 40/50), while squamous cell carcinoma accounted for 20% (10/50). Inoperable cases comprised 88% (44/50), while 12% (6/50) were operable. For tumor stage, 42% (21/50) were classified as tumor stage I–II, and 58% (29/50) as tumor stage III–IV. Chemotherapy was administered to 78% (39/50), while 22% (11/50) did not receive chemotherapy. Radiotherapy was given to 46% (23/50), while 54% (27/50) did not receive radiotherapy. During follow-up, recurrence was observed in 28% (14/50), while 72% (36/50) had no recurrence. Distant metastasis was identified in 28% (14/50), while 72% (36/50) had no metastasis. At the last evaluation, 28% (14/50) had died, and 72% (36/50) were alive at follow-up.

**Figure 1:**
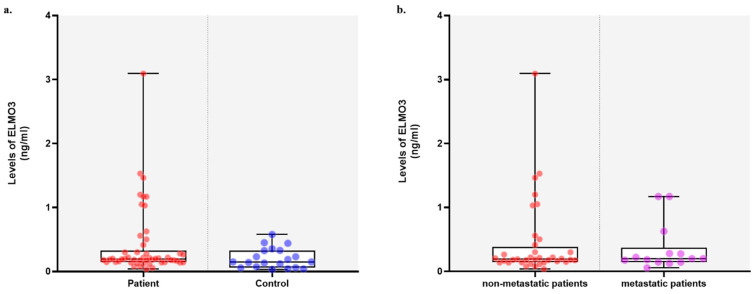
Distribution of serum ELMO-3 protein levels among study groups. (**a**) Box-and-scatter plots show ELMO-3 expression levels in NSCLC patients and healthy controls. Patients were further stratified into low- and high-ELMO-3 expression groups according to the mean value observed in the patient cohort. Similarly, healthy individuals were categorized based on the same cutoff value. (**b**) ELMO-3 levels were compared between metastatic and non-metastatic patients. Comparisons between groups were performed using the Mann–Whitney U test, and no statistically significant differences were observed (*p* > 0.05). Horizontal lines within boxes indicate median values, boxes represent the interquartile range (IQR), and whiskers indicate the minimum and maximum values.

The distribution of ELMO-3 expression levels was evaluated (Figure 1). The mean ELMO-3 level was 0.409 ± 0.543, with a median of 0.195 (0.036–3.092). Based on the defined mean value, patients were classified into low- and high-ELMO-3 level groups. Seventy-six percent of patients (38/50) were categorized as having low ELMO-3 levels, while 24% (12/50) were classified as having a high ELMO-3 level. Protein levels were also evaluated in 20 healthy individuals. The mean protein level was 0.206 ± 0.158, with a median of 0.1495 (range: 0.033–0.577). When the protein levels of healthy individuals were classified according to the mean value observed in patients, high protein levels were detected in 15% (3/20) of individuals, while 85% (17/20) were classified as having low protein levels. Descriptive statistics were presented as mean ± standard deviation for descriptive purposes, while median values were also reported where appropriate. Since ELMO-3 levels were not normally distributed (*p* < 0.05), non-parametric tests (Mann–Whitney U and Kruskal–Wallis tests) were used for group comparisons.

To further investigate the relationship between ELMO-3 levels and clinicopathological characteristics, patients were stratified into low- and high-ELMO-3 groups according to the median serum ELMO-3 level, which was used as a threshold value (Table 1). No statistically significant associations were detected between ELMO-3 status and all clinicopathological parameters (*p* > 0.05).

The presence of outlier values, particularly in the patient group, has influenced the distribution of ELMO-3 levels and potentially affected mean-based analyses and cutoff determination. Associations between ELMO-3 status according to mean value and categorical variables were evaluated using the chi-square test. For variables containing more than two subgroups, Monte Carlo simulation was applied. Among the analyzed parameters, a significant association was observed only between ELMO-3 status and tumor histopathology (*p* = 0.019). Specifically, low ELMO-3 expression was more frequently observed in adenocarcinoma cases, whereas higher ELMO-3 levels were relatively more common in SCC. In the low-ELMO-3 group, 27 patients had adenocarcinoma and 11 had SCC, while in the high-ELMO-3 group 4 patients had adenocarcinoma and 8 had SCC.

### 3.1. Diagnostic Analysis

ROC curve analysis was performed to evaluate the diagnostic performance of serum ELMO-3 levels in differentiating patient groups and to determine optimal cutoff values. The analysis demonstrated limited discriminative ability, and none of the comparisons reached statistical significance. For the patient versus healthy control comparison, the AUC was 0.618 (95% CI: 0.462–0.774, *p* = 0.125). The optimal cutoff value was identified as 15.45 ng/mL, yielding a sensitivity of 72% and a specificity of 55%. Similarly, for metastatic patients versus healthy controls, the AUC was 0.621 (95% CI: 0.430–0.813, *p* = 0.215). The optimal cutoff value was 16.3 ng/mL, with a sensitivity of 71.4% and a specificity of 55%. When metastatic and non-metastatic patients were compared, the AUC was 0.519 (95% CI: 0.344–0.694, *p* = 0.833). The optimal cutoff value was 18.95 ng/mL, corresponding to a sensitivity of 57.1% and a specificity of 50%. Based on these ROC-derived thresholds, patients were stratified into low- and high-ELMO-3 groups for subsequent analyses.

### 3.2. Survival Analysis

**Figure 2:**
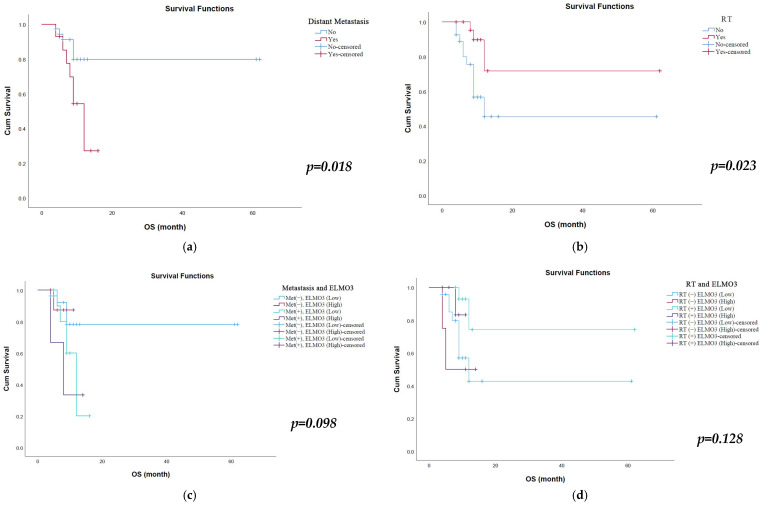
Kaplan–Meier survival curves for overall survival according to clinical parameters. (**a**) Overall survival stratified by distant metastasis status. Patients without distant metastasis showed significantly longer survival than those with metastasis. (**b**) Overall survival according to radiotherapy status. Patients receiving radiotherapy demonstrated significantly improved survival. (**c**) Overall survival according to combined metastasis and ELMO-3 expression status. (**d**) Overall survival according to combined radiotherapy and ELMO-3 expression status.

Kaplan–Meier survival analysis revealed that distant metastasis and radiotherapy status were significantly associated with overall survival. Patients without distant metastasis had significantly longer overall survival compared with those with distant metastasis. The mean OS was 50.9 months (95% CI: 42.90–58.92) in patients without metastasis, whereas it was 10.9 months (95% CI: 8.68–13.09) in patients with distant metastasis (*p* = 0.018) (Table 2, Figure 2a). Similarly, radiotherapy was associated with improved survival outcomes. Patients who received radiotherapy had a significantly longer mean OS of 47.5 months (95% CI: 30.70–64.29) compared with 32.0 months (95% CI: 18.70–45.29) in patients who did not receive radiotherapy (*p* = 0.023) (Table 2, Figure 2b). Other clinicopathological parameters, including age, gender, smoking status, COPD history, ECOG performance status, pathology subtype, tumor size, operability, chemotherapy status, recurrence, and ELMO-3 expression alone, were not significantly associated with overall survival (*p* > 0.05).

Combined subgroup analyses were performed to evaluate the interaction between radiotherapy and ELMO-3 expression as well as metastatic status and ELMO-3 expression on overall survival (Figure 2c,d). Although the differences did not reach statistical significance (*p* = 0.128), patients who received radiotherapy and had low ELMO-3 expression demonstrated the longest survival, with a median OS of 48.9 months (95% CI: 31.65–66.21). Among these patients (*n* = 15), two individuals had survival durations exceeding the median overall survival of 48.9 months. In contrast, patients who did not receive radiotherapy and had high ELMO-3 expression showed markedly shorter survival (9.25 months; 95% CI: 4.58–13.92). Similarly, patients who received radiotherapy but had high ELMO-3 expression also exhibited relatively short survival (10.5 months; 95% CI: 9.61–11.40). In the analysis combining metastatic status and ELMO-3 expression, although the difference was not statistically significant (*p* = 0.098), patients with non-metastatic disease and low ELMO-3 expression had the longest survival (50.2 months; 95% CI: 40.97–59.40). Survival was substantially shorter in patients with non-metastatic disease and high ELMO-3 expression (10.3 months), metastatic disease and low ELMO-3 expression (11.1 months), and metastatic disease and high ELMO-3 expression (8.7 months).

**Figure 3:**
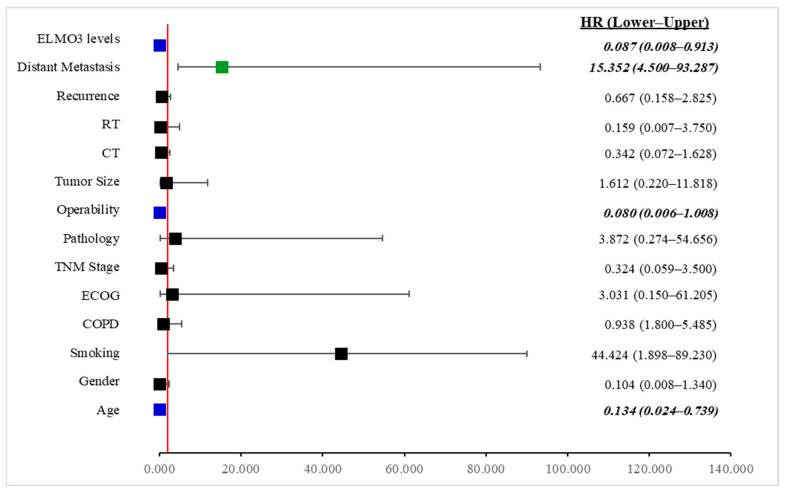
Multivariate Cox regression forest plot of prognostic factors associated with survival. The forest plot illustrates hazard ratios (HRs) and 95% confidence intervals for variables included in the multivariate Cox proportional hazards model. The vertical red line represents HR = 1. Variables located to the right of the line indicate increased risk, whereas those to the left indicate protective effects. Distant metastasis significantly increased the risk of the event, while age, operability, and ELMO-3 levels were associated with reduced hazard. Error bars represent 95% confidence intervals.

A multivariate Cox proportional hazards regression analysis was performed to identify independent prognostic factors associated with survival (Figure 3). The variables included in the model were age, gender, smoking status, COPD, ECOG performance status, type of pathology, tumor size, operability, radiotherapy, chemotherapy, local recurrence, distant metastasis, and serum ELMO-3 levels. The analysis revealed that age, operability, distant metastasis, and ELMO-3 levels were independent predictors of survival. Age was significantly associated with survival outcomes (HR = 0.134, 95% CI: 0.024–0.739, *p* = 0.031). The hazard ratio below 1 indicates that increasing age in the model was associated with a reduced risk of the studied event within the coding scheme used in the analysis. Operability also emerged as a significant protective factor (HR = 0.080, 95% CI: 0.006–1.008, *p* = 0.035), suggesting that patients classified as operable had a markedly lower risk compared with non-operable patients. In contrast, distant metastasis was strongly associated with poorer outcomes and represented the most powerful risk factor in the model (HR = 15.352, 95% CI: 4.500–93.287, *p* = 0.007). Patients with distant metastasis had approximately 15.3-fold higher risk compared with those without metastasis. Importantly, ELMO-3 serum levels were identified as a potential prognostic biomarker (HR = 0.087, 95% CI: 0.008–0.913, *p* = 0.046). Higher ELMO-3 levels were significantly associated with a reduced hazard in the multivariate model, indicating that ELMO-3 may have potential prognostic value in predicting survival outcomes. Although smoking status demonstrated a markedly elevated hazard ratio (HR = 44.424), the association did not reach statistical significance (*p* = 0.072), likely due to the wide confidence interval, suggesting variability within the sample. Similarly, gender, COPD, ECOG performance status, pathology type, tumor size, radiotherapy, chemotherapy, and local recurrence were not significantly associated with survival in the multivariate model (*p* > 0.05). Overall, these findings suggest that distant metastasis is the strongest adverse prognostic factor, while operability and ELMO-3 levels appear to exert protective effects on survival. The identification of ELMO-3 as a potential prognostic biomarker highlights its potential clinical relevance in risk stratification of patients.

**Figure 4:**
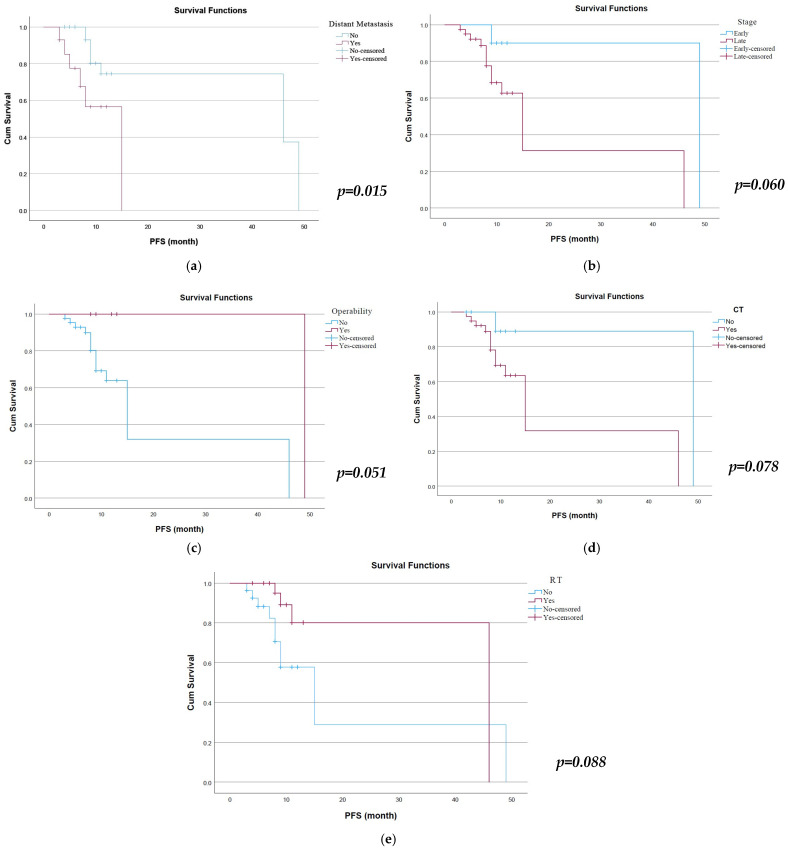
Kaplan–Meier curves for progression-free survival according to clinical parameters such as (**a**) distant metastasis, (**b**) disease stage, (**c**) operability, (**d**) chemotherapy, and (**e**) radiotherapy.

Kaplan–Meier survival analysis was performed to evaluate PFS according to clinicopathological variables (Figure 4). Among the evaluated factors, the presence of distant metastasis was significantly associated with PFS. Patients without distant metastasis demonstrated longer PFS compared with those with metastasis (estimate: 37.7 vs. 10.9 months, *p* = 0.015). Operability status also showed a borderline relationship with PFS, as operable patients exhibited longer survival estimates compared with inoperable patients (49.0 vs. 22.3 months, *p* = 0.051). Furthermore, overall stage demonstrated a trend toward significance, with patients in early stage showing longer PFS compared with late stage (45.0 vs. 22.0 months, *p* = 0.060). In addition, several variables showed borderline associations with PFS. Patients who underwent chemotherapy tended to have shorter PFS compared with those who did not receive chemotherapy (estimate: 22.2 vs. 44.6 months, *p* = 0.078). Similarly, radiotherapy showed a trend toward association with PFS, with patients receiving radiotherapy demonstrating longer survival estimates (38.8 vs. 21.5 months, *p* = 0.088). Overall, distant metastasis emerged as the only variable significantly associated with progression-free survival, while chemotherapy, radiotherapy, operability, and stage demonstrated borderline associations with PFS. Subsequently, a multivariate Cox proportional hazards regression analysis was conducted to determine independent predictors of PFS. The model included age, gender, smoking status, COPD, ECOG performance status, histopathology, stage, operability, tumor size, radiotherapy, chemotherapy, distant metastasis, and serum ELMO-3 levels. In the multivariate model, none of the variables reached statistical significance (*p* > 0.05).

## 4. Discussion

NSCLC has remained one of the most aggressive malignancies worldwide, largely due to late diagnosis and the high frequency of metastatic disease [21]. Therefore, identifying reliable biomarkers associated with tumor progression and metastasis is of major clinical importance. In this study, serum ELMO-3 levels were investigated in patients with NSCLC, and their associations with clinicopathological characteristics and survival outcomes were evaluated. The main findings indicated that serum ELMO-3 levels did not differ significantly between NSCLC patients and healthy controls and were not associated with metastatic status. However, a significant relationship was found between ELMO-3 expression status and tumor histopathology. Furthermore, serum ELMO-3 levels were identified as a potential prognostic factor in multivariate survival analysis. These findings suggest that although circulating ELMO-3 levels may have limited diagnostic value when considered alone, they may still have potential prognostic significance in NSCLC.

ELMO proteins function as regulators of cytoskeletal remodeling and cellular motility, processes that play critical roles in tumor invasion and metastasis. Among this protein family, ELMO-3 has attracted increasing attention because of its potential involvement in cancer progression. Three previous studies [16,17,18] have investigated the role of ELMO-3 in NSCLC. In one study analyzing primary tumors and matched metastatic tissues from NSCLC patients [18], increased ELMO-3 expression was observed in tumors that developed metastases compared with non-metastatic tumors. This elevated expression was associated with hypomethylation of the ELMO-3 gene promoter region, suggesting that epigenetic alterations may contribute to the upregulation of ELMO-3 during metastatic progression. These findings indicate that activation of ELMO-3 might represent an epigenetic mechanism involved in NSCLC metastasis.

Consistent with these observations, another large-scale study [17] including 125 NSCLC patients and 89 healthy controls reported significantly higher ELMO-3 mRNA levels both in tumor tissues and in the serum of patients compared with control groups. In that study, high ELMO-3 expression was found to be significantly associated with larger tumor size, advanced TNM stage, lymph node involvement, and distant metastasis. Additionally, ROC analysis demonstrated strong diagnostic performance, and survival analyses revealed that increased ELMO-3 expression was associated with shorter overall survival. Importantly, ELMO-3 expression was identified as a potential prognostic factor in multivariate analysis. Differences between protein-based measurements and mRNA expression analyses may also contribute to inconsistencies across studies, as circulating protein levels may not directly reflect gene expression levels in tumor tissue.

In addition to clinical observations, experimental studies have provided mechanistic insights into the role of ELMO-3 in NSCLC progression. In other study [16], both ELMO-3 and COX-2 were reported to be significantly overexpressed in NSCLC tissues compared with normal lung tissues. Silencing of the ELMO-3 gene using siRNA was shown to markedly reduce cancer cell proliferation, migration, and invasion in lung cancer cell lines. Furthermore, treatment with COX-2 inhibitors suppressed ELMO-3 expression and reduced tumor growth and metastatic potential in both in vitro and in vivo models. These findings further supported the hypothesis that ELMO-3 may play an important role in tumor progression and may represent a potential therapeutic target.

In contrast to these previous reports, the present study did not identify significant differences in serum ELMO-3 levels between NSCLC patients and healthy individuals or between metastatic and non-metastatic patient groups. Several factors may explain this discrepancy. First, most previous studies focused on ELMO-3 expression in tumor tissues or mRNA levels, whereas circulating protein levels in serum were evaluated in the present study. Alterations observed in tumor tissue expression may not always be directly reflected in circulating protein concentrations. Second, the relatively limited sample size of the current cohort may have reduced the statistical power required to detect subtle differences between groups. Third, differences in tumor heterogeneity, stage distribution, and analytical methodologies may also have contributed to the inconsistent findings across studies.

Despite the absence of diagnostic significance, an important observation of the present study was the identification of serum ELMO-3 levels as a potential prognostic factor in multivariate survival analysis. Kaplan–Meier survival analyses demonstrated that the presence of distant metastasis and radiotherapy status were significantly associated with overall survival. Patients without distant metastasis were found to have markedly longer survival durations compared with those with metastatic disease. Metastasis represents a multistep biological process involving tumor cell invasion, migration, and colonization of distant organs, and therefore reflects an advanced and aggressive stage of tumor biology [22]. Consequently, the shorter survival observed in metastatic patients was consistent with the existing literature.

Longer survival was also observed in patients who received radiotherapy. This finding suggested that radiotherapy may contribute to improved survival outcomes by achieving local tumor control and reducing tumor burden. Radiotherapy has been widely used in the management of NSCLC in both curative and palliative treatment settings, and it has been shown to improve prognosis in appropriately selected patient populations by enhancing local disease control [4,23]. Therefore, the longer survival observed in patients receiving radiotherapy was considered consistent with its known clinical benefits.

In the present study, ELMO-3 expression alone did not demonstrate a significant association with overall survival. This finding suggested that the prognostic impact of ELMO-3 may be influenced by interactions with other components of tumor biology and clinical parameters. Therefore, ELMO-3 may be associated with tumor progression and metastatic potential. In subgroup analyses, although statistical significance was not reached, the longest survival was observed in patients with low ELMO-3 expression who received radiotherapy. This observation may suggest that lower ELMO-3 expression reflects a less aggressive tumor phenotype or may be associated with improved treatment response. Similarly, in analyses combining metastatic status and ELMO-3 expression, the longest survival was observed in patients with non-metastatic disease and low ELMO-3 expression. The absence of significant independent predictors in the multivariate PFS analysis suggests that the clinical utility of ELMO-3 in predicting disease progression may be limited in this cohort.

Multivariate Cox regression analysis allowed the identification of independent prognostic factors affecting survival outcomes. Distant metastasis emerged as the most powerful adverse prognostic factor. The approximately fifteen-fold increase in mortality risk observed in metastatic patients supported the well-established role of metastasis as a key determinant of disease aggressiveness and systemic dissemination in NSCLC. This finding was consistent with previous reports emphasizing the critical role of metastatic disease in determining clinical prognosis and guiding risk stratification in patient management.

Operability was identified as a significant protective factor in the multivariate model. Patients classified as operable demonstrated substantially lower mortality risk compared with non-operable patients. Surgical resection remains the most effective curative treatment option for appropriately selected NSCLC patients, particularly in early-stage disease [24]. Therefore, the findings of the present study supported the established prognostic benefit of surgical eligibility.

Interestingly, ELMO-3 was identified as a protective factor (HR < 1) in the multivariate model. This finding appears inconsistent with its known role in promoting cell motility and invasion and may be related to variable coding or cohort-specific biological characteristics. The lack of a significant association between ELMO-3 and survival in Kaplan–Meier analysis, despite its significance in multivariate Cox regression, may reflect confounding effects or interactions with other clinicopathological variables. Higher ELMO-3 levels were associated with a reduced hazard in the multivariate model, suggesting that ELMO-3 may play a complex role within the tumor microenvironment or tumor biology. Nevertheless, the biological mechanisms underlying this association remain unclear and should be clarified through larger cohort studies and functional investigations.

Age also emerged as a potential factor in the multivariate analysis, indicating that patient-related characteristics may influence survival outcomes. Although smoking status showed a markedly elevated hazard ratio, statistical significance was not reached, likely due to the wide confidence interval and limited sample size.

Overall, the present findings confirm that distant metastasis is one of the most powerful prognostic factors in NSCLC, while operability appears to have a protective effect on survival. Furthermore, the identification of serum ELMO-3 levels as a potential prognostic biomarker suggests that this molecule may have potential clinical utility in risk stratification. However, additional large-scale prospective studies are required to validate the clinical relevance of ELMO-3.

Another noteworthy observation in the present study was the significant association between ELMO-3 expression status and tumor histopathology. Higher ELMO-3 levels were observed more frequently in squamous cell carcinoma than in adenocarcinoma. This finding suggests that ELMO-3 expression patterns may vary across histological subtypes of NSCLC and may reflect differences in underlying molecular pathways. Nevertheless, further studies are required to confirm this observation.

Several limitations of the present study should also be acknowledged. The relatively small sample size may limit the generalizability of the findings. In addition, only circulating serum protein levels of ELMO-3 were evaluated, while tissue expression and genetic or epigenetic alterations were not investigated. The wide confidence intervals observed for several variables in the multivariate analysis suggest potential model instability, which may be due to the limited sample size and the relatively large number of variables included in the model. Future studies integrating serum biomarker analysis with tumor tissue investigations, including gene expression and methylation profiling, may provide a more comprehensive understanding of the role of ELMO-3 in NSCLC biology.

## 5. Conclusions

In summary, this study evaluated serum ELMO-3 levels in patients with non-small cell lung cancer and examined their associations with clinicopathological characteristics and survival outcomes. Although serum ELMO-3 levels did not differ significantly between patients and healthy controls or between metastatic and non-metastatic groups, ELMO-3 expression was associated with tumor histopathology. Additionally, serum ELMO-3 levels were identified as a potential factor in multivariate survival analysis. These findings suggest that circulating ELMO-3 may have a potential role in the prognostic evaluation of NSCLC, although its diagnostic value appears limited. Given the relatively small sample size and the lack of tissue-based molecular analyses, further studies with larger patient cohorts and integrated molecular approaches may help clarify the clinical relevance and biological role of ELMO-3 in NSCLC.

## Figures and Tables

**Table 1:** Evaluation of the association between the median protein levels of ELMO3 and various clinicopathological parameters using the chi-square test.

Parameters		Low	High	*p* Value
Age	<60	11	11	1.000
	>60	14	14	
Gender	Female	2	6	0.123
	Male	23	19	
Smoking	No	4	3	0.684
	Yes	21	22	
COPD	No	18	18	1.000
	Yes	7	7	
ECOG	I–II	23	22	0.637
	III–IV	2	3	
TNM Stage	Early	9	10	0.771
	Late	16	15	
Pathology	SCC	4	6	0.48
	Adeno	21	19	
Operability	No	20	24	0.082
	Yes	5	1	
Tumor Stage	I–II	9	12	0.39
	III–IV	16	13	
CT	No	15	12	0.395
	Yes	10	13	
RT	No	5	6	0.733
	Yes	20	19	
Recurrence	No	18	18	1.000
	Yes	7	7	
Distant Metastasis	No	19	17	0.529
	Yes	6	8	

CT: Chemotherapy, RT: Radiotherapy, COPD: Chronic Obstructive Pulmonary Disease, ECOG: Eastern Cooperative Oncology Group, SCC: Squamouse Cell Carcinoma.

**Table 2:** Kaplan–Meier survival analysis of overall survival according to clinicopathological and treatment-related parameters.

Parameters		Estimate	Std. Error	95% CI		*p* Value
				Lower	Upper	
Age	<60	39.808	6.400	27.265	52.351	0.351
	>60	37.754	8.272	21.540	53.967	
Gender	Female	9.813	0.746	8.350	11.275	0.759
	Male	39.905	5.668	28.797	51.013	
Smoking	No	9.800	0.179	9.449	10.151	0.565
	Yes	38.949	5.598	27.977	49.922	
COPD	No	35.241	6.847	21.822	48.661	0.418
	Yes	13.883	1.105	11.718	16.048	
ECOG	ECOG I–II	38.587	5.906	27.011	50.164	0.677
	ECOG III–IV	11.667	1.089	9.533	13.800	
TNM Stage	Early	44.667	13.336	18.528	70.805	0.087
	Late	37.314	5.847	25.853	48.774	
Pathology	SCC	36.463	8.860	19.097	53.830	0.698
	Adeno	41.009	6.865	27.552	54.465	
Operability	No	38.738	6.284	26.420	51.055	0.257
	Yes	48.750	10.609	27.957	69.543	
Tumor Size	T I–II	45.597	7.349	31.192	60.001	0.118
	T III–IV	33.744	8.006	18.052	49.436	
CT	No	40.970	12.626	16.223	65.716	0.337
	Yes	38.680	5.919	27.079	50.282	
RT	No	31.992	6.784	18.696	45.288	0.023
	Yes	47.496	8.570	30.698	64.294	
Recurrence	No	11.190	0.514	10.182	12.199	0.977
	Yes	41.462	7.207	27.336	55.587	
Distant Metastasis	No	50.912	4.087	42.901	58.923	0.018
	Yes	10.887	1.125	8.682	13.092	
ELMO-3 Status	Low	39.090	6.060	27.212	50.968	0.717
	High	11.723	1.141	9.487	13.959	

CT: Chemotherapy, RT: Radiotherapy, CI: Confidence Intervals, COPD: Chronic Obstructive Pulmonary Disease, ECOG: Eastern Cooperative Oncology Group, SCC: Squamouse Cell Carcinoma

## Data Availability

The original contributions presented in this study are included in the article. Further inquiries can be directed to the corresponding author.
